# The stages of implementation completion for evidence-based practice: protocol for a mixed methods study

**DOI:** 10.1186/1748-5908-9-43

**Published:** 2014-04-05

**Authors:** Lisa Saldana

**Affiliations:** 1 Oregon Social Learning Center, 10 Shelton-McMurphey Blvd., Eugene, OR 97401, USA

**Keywords:** Stages of implementation, Implementation costs, Implementation processes, Milestones

## Abstract

**Background:**

This protocol describes the ‘development of outcome measures and suitable methodologies for dissemination and implementation approaches,’ a priority for implementation research. Although many evidence-based practices (EBPs) have been developed, large knowledge gaps remain regarding how to routinely move EBPs into usual care. The lack of understanding of ‘what it takes’ to install EBPs has costly public health consequences, including a lack of availability of the most beneficial services, wasted efforts and resources on failed implementation attempts, and the potential for engendering reluctance to try implementing new EBPs after failed attempts.

The Stages of Implementation Completion (SIC) is an eight-stage tool of implementation process and milestones, with stages spanning three implementation phases (pre-implementation, implementation, sustainability). Items delineate the date that a site completes implementation activities, yielding an assessment of duration (time to complete a stage), proportion (of stage activities completed), and a general measure of how far a site moved in the implementation process.

**Methods/Design:**

We propose to extend the SIC to EBPs operating in child service sectors (juvenile justice, schools, substance use, child welfare). Both successful and failed implementation attempts will be scrutinized using a mixed methods design. Stage costs will be measured and examined. Both retrospective data (from previous site implementation efforts) and prospective data (from newly adopting sites) will be analyzed. The influence of pre-implementation on implementation and sustainability outcomes will be examined (Aim 1). Mixed methods procedures will focus on increasing understanding of the process of implementation failure in an effort to determine if the SIC can provide early detection of sites that are unlikely to succeed (Aim 2). Study activities will include cost mapping of SIC stages and an examination of the relationship between implementation costs and implementation performance (Aim 3).

**Discussion:**

This project fills a gap in the field of implementation science by addressing the measurement gap between the implementation process and the associated costs. The goal of this project is to provide tools that will help increase the uptake of EBPs, thereby increasing the availability of services to youth and decreasing wasted resources from failed implementation efforts.

## Background

Although many evidence-based practices (EBPs) have been developed, large knowledge gaps remain regarding how to routinely move EBPs into usual care [1]. There is much still to be learned about the key processes necessary for successful implementation, including the steps that are essential to effectively transport EBPs into usual care, and how to measure if they have occurred well [2].

Efforts to examine implementation outcomes are hindered by the lack of standardized tools to measure the key processes and stages of implementation [3]. For example, no standardized method exists to measure or predict what early implementation activities are necessary for successful program start-up, or to estimate the costs and resources necessary to complete implementation over and above the cost of the EBP itself [4], limiting the accuracy of fiscal decision-making about total implementation costs. Such limitations impede the ability to develop interventions/strategies to enhance and support successful real-world implementation efforts.

With the increased focus and effort to implement EBPs in real-world community settings [5] comes recognition of the complexity of the task, which involves planning, training, quality assurance, and interactions among developers and system leaders, front line staff, and consumers [1]. It is generally thought that it takes a site a minimum of two years to complete the implementation process [6] and that achievement is strongly influenced by the success of the implementation methods [7]. However, little is known about which aspects of these methods are most important for successful implementation [8]. Recently, there has been increased focus on understanding what steps in the implementation process are essential to effectively transport EBPs [2,9].

**Figure 1:**
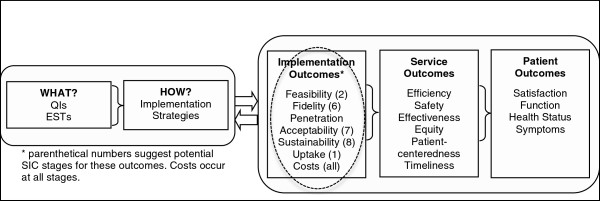
**Conceptual model for implementation research (adapted from Proctor et al. **[3]**) Circled area shows target of proposal.**

There is consensus that implementation is likely a recursive process with well-defined stages that are not necessarily linear and that impact each other in complex ways [6,10]. A treatment developer or purveyor typically assists programs in navigating their way through each of the implementation stages in an effort to ensure that program elements are delivered in the manner intended. Researchers have called for the measurement of the key processes and stages of adopting an EBP, and the assessment of the fidelity of implementation methods [11,12]. As shown in Figure 1[11], implementation research includes three distinct levels of evaluation including patient, service, and implementation outcomes. Leading services researchers have noted the need for models, like that provided in Figure 1, to link key implementation stages and outcomes to service and patient outcomes [13,14]. To date, limited tools are available to inform distinctions between these key levels of outcomes, thus limiting the evaluation of these discrete processes.

The current protocol describes a study in which we seek to narrow this gap by focusing on further development of a measure (*i.e*., the Stages of Implementation Completion; SIC [15]). Through this study, we will examine the generalizability of the SIC across four EBPs in different service sectors serving child and family mental health needs. A glossary of terms is provided in the subsection below.

Glossary of terms used throughout the protocol

**Practice:** A given specific EBP.

**Site:** Newly adopting programs for each EBP.

**Phases:** Pre-Implementation, Implementation, and Sustainability.

**Stages:** The 8 Stages of Implementation on the SIC ranging from Engagement to Competency.

**Universal Items:** Implementation activities identified on the SIC that are common across the EBPs.

**Unique Items:** Implementation activities identified on the EBP-specific SIC that are unique to that EBP.

**Retrospective:** Data from sites that previously adopted the respective EBP prior to project.

**Prospective:** Data from sites that attempt to implement the EBP during the grant period.

### Considerations for measuring implementation

#### Progression and timing

Given the non-linear yet staged progression of implementation, the measurement of this process must be flexible enough to capture potential variations. Having a well-defined system of implementation and knowledge about the typical successful progression through the stages could increase the likelihood that a purveyor could provide sites with information in the early stages that will help to support their success in later stages [8] in order to help realistically assess and calibrate their efforts either to proceed, or reconsider whether or not their current implementation plan remains viable. Ideally, such a measure could indicate when agencies are starting to falter or are on an unsuccessful path. Determining when specific barriers hinder successful outcomes is crucial for understanding the implementation process. It would be useful to have early signals that such barriers are on the horizon, and to know what distinguishing factors make these barriers significant enough to prevent moving forward (*e.g*., site and/or fiscal considerations).

#### Learning from failures and success

Although organizational factors such as climate and culture have repeatedly demonstrated influence on successful implementation [16], less is known about when failures occur in the implementation process and what the site’s perceptions are as to why these failures occurred. Much can be learned from sites that initiate the implementation of an EBP and then fail to reach milestones such as start-up or sustainability [17] in addition to those that are successful. Until recently, studies recruited sites retrospectively [11] or used recruitment designs where EBP champions recruited sites to implement [7], therefore not reflecting real-world conditions in which sites often initiate the process. We propose a naturally occurring design in which sites self-select to adopt a practice. This allows for prospective observation of the specific barriers that are perceived by sites as insurmountable at particular stages in the implementation process, and the differences between sites that are and are not able to overcome them. We will evaluate if barriers that result in failure can be detected using a standardized measure.

#### Understanding the costs of implementation and when they accrue

Ritzwoller and colleagues [4] argued for the need for standardized methods of analysis of cost data for behavioral medicine implementation and suggested that this gap in knowledge might play a key role in why new interventions fail to translate from research to practice. Understanding cost is complex and difficult to estimate partly because such estimations depend on what phase of implementation the site is engaged in [3]. Related and important to communities, are the specific activities in the implementation process that are necessary (versus optional) for program success, and the resources required to complete them. Although leading theories and frameworks include conceptualization of implementation costs as an important factor (*e.g*., Figure 1), such costs are an understudied aspect of implementation science [18]. Using the SIC, we developed a standardized method for estimating implementation costs per stage that has the potential to generalize across EBPs and sites [19]. This could be of high value for decision-makers who are responsible for determining if they can afford to adopt new practices; some decision-makers might underestimate the resources needed for implementation, while others might over-estimate the needed resources and limit themselves from adopting practices that could be beneficial to their communities.

#### The Stages of Implementation Completion (SIC)

The SIC is an eight-stage assessment tool developed as part of a large-scale randomized implementation trial [15]. The trial contrasted two methods of implementing an EBP for youth with serious behavioral problems in the juvenile justice and child welfare systems, Multidimensional Treatment Foster Care (MTFC), in 53 sites in California and Ohio [20]. As shown in (Additional file 1), the SIC has eight stages with sub-activities within each stage. The stages range from Engagement with the developers to practitioner Competency and map onto three well-accepted phases of implementation (Pre-Implementation, Implementation, Sustainability) [2].

#### Measuring multiple levels

One of the complexities of implementation is that different agents are involved over time (Additional file 1). Initially, system leaders (*e.g*., juvenile justice, mental health, school) are involved in the decision of whether or not to adopt an EBP, and they often assess implementation feasibility. Over time, the key players in the process shift from system leaders to agency leaders and practitioners, and clients receiving services. A measure of implementation must incorporate data from these multiple levels if it is to accurately capture the complexity at various points in the process.

#### SIC scores

Two scores are calculated for each SIC stage. First, the amount of time that a site spends in a stage is calculated by date of entry through date of final activity completed (*i.e*., Duration Score). Because the implementation process is nonlinear, the Duration Score takes into account that activities might not be completed sequentially within a stage. Second, the percentage of activities completed within a stage is calculated (*i.e*., Proportion Score). A site might quickly complete a stage, but not complete all of the activities within that stage. Including both the Duration and Proportion Scores allows for an evaluation of which of these (and the interaction) is most important for successful implementation, and if these findings differ by stage. A third score identifies the final stage achieved (Stage Score).

#### Predictive ability of scores

Saldana and colleagues [21] found that SIC scores predicted variations in implementation behavior for sites attempting to adopt the MTFC model. Based on stage Proportion and Duration Scores, sites were accurately identified (*i.e*., face validity) through agglomerative hierarchical cluster analyses. Employing Cox proportional hazard models, cluster membership was used to predict successful MTFC start-up. Successful program start-up was predicted by site performance, as measured by the SIC in the first three stages (*i.e*., predictive validity). Sites that completed implementation activities thoroughly (high proportion) but relatively rapidly (duration) were most likely to initiate service; sites that took longer to complete each stage and completed fewer activities had a significantly lower hazard of having their first placement within the study period (HR = 0.190, p = 0.01) than rapid and thorough completers. Implementation condition did not significantly contribute to this model (p = 0.33), suggesting that prediction using the SIC was not attributable to implementation strategy.

#### Mapping cost by stage

Another analysis from the same trial [19] demonstrated that the SIC could serve as a map for costing implementation procedures. Procedures included calculations of fees, expenses, and person hours necessary to complete each Stage. Differences in cost structures (*i.e*., the when and how much for resource allocation) were identified between the implementation conditions despite implementation of the same intervention (*i.e*., MTFC). Differences in costs occurred primarily during the pre-implementation phase. Patterns of resource allocation were identified; although some stages were less expensive for one strategy than the other, the less expensive strategy might require more person hours or effort. Such information is critical for decision-makers when determining resource allocation and viability of the implementation strategy.

#### Proposed adaptations of the SIC

Similar to the core components proposed by Blasé and Fixsen [14] as being essential for successful implementation, each of the eight stages describes key milestones that are necessary (*e.g*., completion of training). We propose to adapt activities within each stage to target the specific tasks necessary to complete implementation. Some activities are expected to vary by EBP, while others are expected to be common/universal, such as fidelity monitoring. Our previous work showed that the decision to implement a new practice is largely based on the community need [22], and this perception is likely influenced by what service sector is accessing the population. This study will sample EBPs in three service sectors (school, substance abuse, juvenile justice) and combine this data across the original EBP in child welfare to build hypotheses about the differences in implementation processes between four sectors.

#### Potential impact to the field

As noted, there is a dearth of standardized measurement of implementation process, milestones, and costs. This gap impedes efforts to help inform real world implementation efforts for both researchers and EBP adopters. Measures such as the SIC that can increase the understanding of implementation processes that relate to the successful adoption of EBPs are needed. Such measures have the potential to contribute to the development of implementation interventions designed to improve success rates for sites at-risk for faltering or discontinuing implementation efforts. The goals of this project are to extend the SIC to additional EBPs in key child and family service systems, to measure meaningful implementation outcomes, and to examine the potential generation of universal items across EBP implementation strategies. We propose a mixed methods strategy to increase understanding of what the SIC scores represent to end users and to researchers. We also will evaluate a strategy for mapping implementation resources by stage that could inform the creation of user friendly fiscal plans for implementation efforts.

### Preliminary studies

#### MTFC-SIC for real-world MTFC sites

To evaluate the potential of the SIC to be utilized in a non-research context, the MTFC-SIC was examined with real-world, usual implementation MTFC sites. Because the measure was developed as part of a research trial and sites were recruited, we wanted to test the measure with non-study sites to ensure that the SIC could be used to adequately measure implementation performance and outcomes outside of the context of a controlled research design. In collaboration with the MTFC purveyor organization, records from the 75 most recently implemented MTFC sites were examined retrospectively. Using the MTFC-SIC, activity completion dates were recorded. We discovered that the SIC was not as useful for sites with existing programs that were adding additional MTFC teams to their organization; expansion sites did not complete the implementation process as thoroughly as those sites that were newly initiating their first program (due to previously completing the full process with their first program). Therefore, we limited the data to 35 newly adopting sites. Outcomes from the MTFC implementation research trial were replicated. Sites were successfully clustered into groups and these clusters were used in a Cox proportional hazard survival model. Sites that took longer to complete their pre-implementation stages had a greater risk of discontinuing than those who completed their stages more rapidly (hazard ratio [HR] = 26.50, p-value < 0.002). In addition to demonstrating the reliability of SIC scores in describing implementation performance and outcomes, this preliminary work illustrates the ability for a purveyor to complete the SIC based on retrospective records of usual implementation activities.

#### MTFC-SIC measurement properties

The measurement properties of the MTFC-SIC also were evaluated. Several challenges for evaluating its psychometric properties were considered (*e.g*., scores include both dichotomous and time variables, meaning of missing data). Given these challenges, the reliability and validity of the MTFC-SIC items [23,24] were evaluated using IRT-based Rasch models [25]. Proportion items and Duration items were evaluated using dichotomous and Poisson [23] models [24,26] and their multilevel extensions (HLM) [27]. Psychometric analysis suggested that the SIC was both a valid and reliable measure of the implementation process for MTFC. For a full description of the challenges and psychometric outcomes, see Additional file 2.

## Methods

Although the primary aims of the described study are to determine if the SIC can be applied across EBPs from different service sectors and accurately predict successful implementation outcomes, data provided from this project will allow investigation of EBP-specific implementation outcomes. Analyses will include examination of the impact that site demographic characteristics have on implementation behavior and cost outcomes. Moreover, because of the stage independence of the SIC, the varying impact of behavior in different stages will be evaluated.

### Participating evidence-based practices

The developers of three widely implemented EBPs from three public service sectors serving children and families agreed to participate in the current project: Multisystemic Therapy (MST) from the juvenile justice sector [28], Multidimensional Family Therapy (MDFT) from the substance abuse sector [29], and a computerized version of Coping Cat (CC) from the school setting [30,31]. All three are recognized in the National Registry of Evidence-Based Programs and Practices (http://www.nrepp.samhsa.gov) and, similar to MTFC, use an ecological approach to address clinical needs for children and families. MST and MDFT are intensive community-based interventions for serious externalizing behaviors, whereas CC is an office-based EBP for severe anxiety. Although there are many EBPs, the selection criteria for inclusion in this study were: (a) EBP for child and family mental health within key service sectors (schools, juvenile justice, substance use) similar to the original model (in foster care); (b) large real-world uptake within the respective service sectors in order to conduct study procedures; and (c) EBP developers who expressed interest in using the SIC and in advancing understanding of the universal implementation elements shown to increase the successful uptake of EBPs.

The recruited EBPs all have a large implementation footprint for the service sectors in which they are adopted. MST purveyors have implemented MST in 38 states and 13 nations; MDFT across 11 states and 6 countries; and CC across the U.S. and 3 countries. All three EBPs report a strong likelihood of continued growth.

### Specific aim 1: adaptation and extension through retrospective and prospective analysis

**Figure 2:**
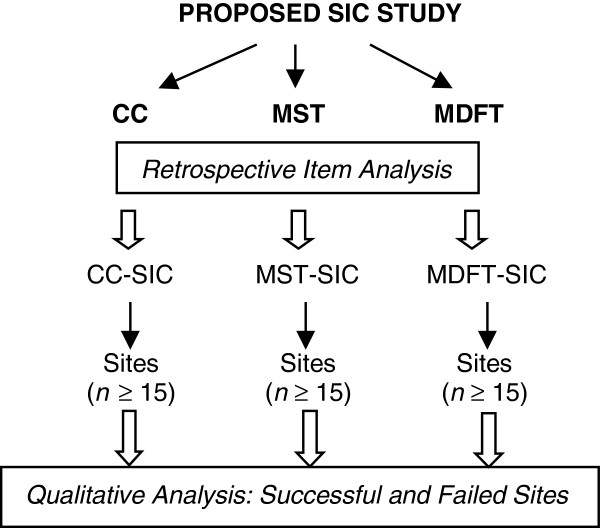
Diagram of study procedures.

Figure 2 provides a diagram of the overall study procedures. The start-up for each EBP was rolled-out over the first year. An initial one-day site visit was conducted with each of the developer/purveyor organizations to learn about the standard implementation process for the EBP. The PI and developers operationalized implementation activities and defined completion criteria for these activities. From this initial adaptation work, EBP-specific and universal activities within each stage currently are being mapped onto the SIC Stages.

### Retrospective data mapping

#### Mapping

Each of the participating EBPs agreed to provide data related to previous sites that have already implemented the EBP, and this data collection is underway. Research assistants from each of the EBPs were trained by the PI to code retrospective data related to a minimum of 15 formerly implemented sites. This number of sites was selected because it balances time and other resource demands, is sufficient for observing variability in implementation activities, and is sufficient for conducting preliminary Rasch-based measurement models.

#### Modification

Based on an initial examination of how well activities appear to define the stages, modifications will be made to each version of the SIC as needed. This might include moving activities from one stage to another, deleting activities, or redefining them. This iterative process has occurred for all three of the EBPs, with further modifications expected at the completion of data analyses. If substantive modifications are needed, an additional 15 sites will be coded retrospectively to verify that any modifications improve the measure’s ability to accurately measure implementation. Collaboratively with the developers, a final EBP-specific SIC will be determined for use in prospective data collection.

#### Prospective data collection

Research staff at the EBP organizations will be trained on data collection procedures for all newly adopting sites. A point person has been identified at each organization to monitor when new sites contact the purveyor to consider implementing the EBP (Stage 1) and to begin tracking activity data on the sites. The purveyors will be trained to note the completion of implementation activities by sites and the resources (both fees and person hours) needed to complete them. Data will include site demographics of all new sites [32]. To ensure reliability of data collected, within each purveyor organization, both the data collection assistant and the EBP site coordinator will independently record 10% of data.

### Analytic plan

#### Scoring

Recalling that the implementation process is known to be nonlinear, the scoring of the SIC does not rely solely on chronological completion of activities or stages. It also does not assume that all sites will complete all activities in a stage or within a given timeframe. As described previously, each site will be given stage Proportion and Duration scores, and a Final Stage score.

#### Measurement properties

Both retrospective and prospective data will be evaluated using IRT-based measurement models (*i.e*., dichotomous & Poisson Rasch models for activity completion and time-to-activity completion) and software (*i.e*., WINSTEPS, FACETS, HLM). Four sets of analyses will be conducted: (i) retrospective, (ii) prospective, (iii) combined retrospective and prospective, and (iv) universal/combined across EBPs. To thoroughly assess the reliability and validity characteristics of each EBP-SIC version, psychometric analyses will evaluate activity and site distributions, reliability, fit, variance components, and dimensionality (*i.e*., replication of methods described in Additional file 2).

A critical assumption is that item difficulty estimates are stable across uses of an instrument [33]. Thus, the activity estimates from retrospective data will be compared to those from prospective data, with stable estimates supporting the EBP-SIC versions. Invariance of activities ‘common’ across EBPs will be evaluated. Assuming invariance, common item equating [34] will allow simultaneous calibration across EBPs. Multilevel formulations will provide activity, stage and site estimates for each EBP. Likewise, an IRT-based item bifactor model [35] will provide loadings for each activity on its given stage and on a general implementation dimension.

#### Power

For IRT models, statistical power refers to the precision of parameter estimates and standard errors. Based on Linacre [36], the smallest number of activities and sites (*i.e*., retrospective data) are sufficient for estimates accurate within ±1 logit, with the prospective (and combined) data affording even greater precision. The combined data also are sufficient for accurate estimation of multilevel Rasch models [37].

#### Implementation performance: hypothesis testing

Replicating procedures from the original MTFC implementation trial, agglomerative hierarchical clustering methods will be employed to find similarities within sites from each of the EBPs with regard to both Proportion and Duration scores for each stage. Sites will be clustered using each of these variables independently, as well as together. Cox proportional hazard survival models will be conducted with days-to-event as a time to event outcome for each of the three sets of clusters. The events analyzed will include significant milestones such as program start-up and certification. Additional significant events will be determined in collaboration with EBP developers in order to ensure that the SIC is able to accurately predict milestones that are meaningful to the developers. It is hypothesized that each SIC version will produce outcomes similar to those found for MTFC such that both Proportion and Duration are significantly related to successful implementation outcomes [21]. The appropriate rate and necessary proportions might differ between EBPs and service sectors. Universal items will be evaluated to determine if they are more critical than unique items for successful implementation. For any analyses that combine retrospective with the prospective data, this characteristic will be included as a covariate to evaluate any differences.

### Specific aim 2: early detection of potential failure by increasing understanding of the underlying SIC mechanisms

#### Participants

A random sample of sites from each EBP that discontinue the implementation process at any point from Engagement to Competency, or that successfully achieve competency, will be recruited for participation. EBP developers estimated that eight sites across EBPs will discontinue each year. Identified sites will be asked to consent to participate in a one-time semi-structured qualitative interview using video-chat technology*.* Participants will be compensated for their time. Consenting procedures will include a request for use of the sites’ SIC data in relation to their identified data.

#### Demographics and position characteristics

Basic personal demographics (*e.g*., age, gender, education) and position characteristics (*e.g*., time in position, position level) will be assessed. Participants will be recruited from each site that was involved in the implementation effort such that programs that discontinued early on in the process might only have administrative-level participants, whereas programs that had undergone training might also have clinician-level participants.

#### Interview

Using an interview guide similar to those employed in previous studies of barriers and facilitators of EBP implementation [38,39], video-chats will be conducted by the PI and a trained qualitative research team. Interviews are anticipated to last between 1 and 1.5 hours and will utilize techniques described in detail elsewhere [40-43]. All video-chats will be digitally recorded and transcribed.

Participants will be asked a series of questions designed to elicit information on knowledge, attitudes and behavior related to the EBP, characteristics of the agency and team, the external environment, and the implementation process. For example, depending on how far they progressed in the implementation process, informants will be asked: (a) how they were involved in implementing the EBP; (b) what they know about the EBP philosophy and procedures; (c) how effective the planning process was in the pre-implementation phase (Stages 1 to 3); (d) how effective the training was in building skills in the EBP; (e) whether they believe the EBP would be helpful to the target population; (f) whether the use of EBPs changed/would change their usual pattern of service delivery; (g) whether the EBP required changes in existing agency policies or procedures; (h) what costs and benefits were encountered in using the EBP; and (i) suggested changes for the EBP to have been successfully implemented.

### Analytic plan

#### Qualitative analysis

As described by Palinkas and colleagues (2011), data from qualitative interviews will be coded for analyses [44]. Using a methodology of ‘Coding Consensus, Co-occurrence, and Comparison’ outlined by Willms [45] and rooted in grounded theory (*i.e*., theory derived from data and then illustrated by characteristic examples of data) [46], interview transcripts will be analyzed. First, investigators will prepare short descriptive ‘memos’ to document initial impressions of topics and themes and their relationships and to define the boundaries of specific codes (*i.e*., the inclusion and exclusion criteria for assigning a specific code) [47]. Then, the empirical material contained in the transcripts will be independently coded by the project investigators to condense the data into analyzable units. Segments of text ranging from a phrase to several paragraphs will be assigned codes based on *a priori* (*i.e*., from the interview guide) or emergent themes (also known as open coding) [48]. Codes will be assigned to describe connections between categories and between categories and subcategories (*i.e*., axial coding) [49]. Each text will be independently coded by at least two investigators. Disagreements in description of codes will be resolved through discussion between investigators and enhanced definition of codes. The final list of codes will consist of themes, issues, accounts of behaviors, and opinions that relate to implementation. Two investigators then will separately review transcripts to determine the level of agreement in the codes applied; agreement ranging from 66 to 97 percent depending on level of coding (general, intermediate, specific) indicates good reliability in qualitative research [50]. Based on these codes, the software QSR NVivo [51] will be used to generate a series of categories arranged in a treelike structure connecting text segments grouped into separate categories or ‘nodes’. These nodes and trees will be used to examine the association between different *a priori* and emergent categories and to identify the existence of new, previously unrecognized categories. Finally, the different categories will be further condensed into broad themes using a format that places implementation failures within the framework of the organizational and system characteristics [46]. The themes and their relationships to one another then will be organized to create a heuristic model of implementation process that can be used to develop and test hypotheses related to underlying processes of the SIC.

#### Mixed methods analysis

Data from discontinued sites’ SIC assessment, as well as their site characteristics will be examined in relation to qualitative outcomes to help increase understanding of how site behavior, as defined on the SIC, relates to on-the-ground decision-making using the mixed method technique of ‘convergence’ [44, 46, 49]. This will allow for an assessment of the SIC’s utility in providing ‘early signal detection’ of potential problems impeding successful implementation (*e.g*., does longer duration in pre-implementation most often indicate that leadership does not have an accurate understanding of how to accomplish a key implementation activity?). Thus, qualitative data will help to inform if there are reliable patterns of behaviors that are linked to particular patterns of SIC scores, thereby helping to unpack the potential for SIC scores to serve as a proxy for less easily observed phenomenon.

## Specific aim 3: costs by stage and implementation outcomes

### Cost mapping utilizing the SIC

#### Data collection

At the initial adaptation meeting, an assessment of the developers/purveyors’ perceptions of effort, resources and fees associated with each of the identified activities was conducted. Throughout prospective data collection procedures, data will be collected regarding the estimated person hours and resources used by sites to complete the SIC activities.

#### Additional costing procedures

Although resources such as person hours and fixed fees will be identified through the data collection calls, additional efforts will be necessary to cost implementation activities. Wherever possible, cost components such as wages and travel costs will be based on national average estimates in order to maximize the generalizability of the results and minimize the potential that performance sites are not typical with respect to salary structures. National public databases, such as the Bureau of Labor Statistics, will be accessed to determine cost estimates for variables such as travel expenses, salaries, and meeting expenses for activities related to implementation.

### Analytic plan

#### Cost estimates

There are three varieties of costs to measure within each stage of the SIC to evaluate the cost of implementation. First, there are direct costs of the implementation services to the site, which generally consist of fees charged by the EBP purveyor. Second, there are indirect costs of site personnel time that is spent conducting the implementation (*i.e*., doing things that do not directly produce client services, and which will not be necessary once implementation is complete). Third, there are ancillary costs, which are made up of the actual infrastructure investments that are required for implementation (*e.g*., the costs of installing a new IT system for fidelity monitoring).

Direct costs will be measured as invoices from the EBP vendor to the site for services and support provided in each stage of the SIC. Indirect and ancillary costs will be measured using a time/resource log designed as part of the project. Each EBP will require a slightly different tool that will depend on the specific nature of the intervention, and will assess personnel (*e.g*., therapists, administration) and other costs that pertain to the implementation but not to the provision of clinical services to clients. We will collect unit cost (such as personnel time), and to the extent possible, use national data to generate the cost weights so that the estimated indirect costs will be representative of what a randomly selected program could expect.

Costs of implementation will be expressed as average cost per SIC stage for each site. However, the raw average cost will not describe the range of possibilities. For example, a site that takes longer to move through an SIC stage will probably have a higher cost for that stage, and merely including it in an average cost calculation would be misleading. Consequently, regression techniques will be used to estimate risk-adjusted cost functions, which can vary with such factors as the number of clients treated by a program or the time required to move through each stage of the SIC. The dependent variable of the regression will be the logged implementation cost for each stage for each site. Ranges of costs will be imputed using the regression models, where staged implementation costs, and 95% confidence intervals for those costs, will be imputed by applying the Duan smearing estimate to predict the levels of SIC stage costs from the logged cost regression.

#### Implementation performance and cost

Using regression models for each EBP, variations in cost will be examined as they relate to SIC scores. Assumedly, longer Duration scores will increase resource allocation needs, but perhaps not significantly. The costs that are mapped onto activities will be calculated in relation to individual activities as well as stages. Cost curves will be estimated to help inform the optimal rate and Proportion of activity completion for sites to achieve success. It might be the case that sites that have higher initial costs (*e.g*., due to a high Duration) are more likely to have low Proportion scores during later stages (*i.e*., attempting to reduce implementation costs downstream). These variations will be considered in relation to successful achievement of implementation milestones.

### Trial status

This protocol has been underway for approximately one year. The study protocol has been approved by the Institutional Review Board. All site visits and initial adaptations have taken place, with each adaptation having gone through several revisions. MST and MDFT are nearing completion of their retrospective data collection, whereas the CC program is well into prospective data collection. Data collection tools for the next study period focused on prospective data collection across all sites are under development, including the cost and resource logs specific for each practice. Current efforts are underway to identify universal items across each of the three adaptations compared to the original MTFC-SIC.

## Discussion

During the final year of study procedures for each EBP, developers will be collaboratively involved in determining the most beneficial uses of the SIC. All EBP-specific outcomes will be shared, and the way that the SIC operates for each EBP will be explained to the developers. For example, the original MTFC-SIC outcomes indicated that sites that linger too long in the pre-implementation phase are less likely to achieve successful program start-up; the EBP developers/researchers might use this information to inform future modifications to their implementation process such as developing an enhanced protocol for struggling sites. Similarly, the developers/purveyors might determine that such information is important feedback for sites as they decide if it is in their best interest to proceed or not. In order for the EBPs to benefit from the SIC, a full understanding of the scoring methods, interpretation, and potential utility must be conveyed.

### Innovation

We believe that the proposed work is innovative in three primary ways.

### Generalization

Adopters, EBP developers, and researchers could all benefit from having a measure that helps determine early on and throughout the implementation process if sites are doing well, doing poorly, or just ‘getting by.’ For adopters, such information would allow for ongoing progress monitoring and could inform decisions about potential corrections. For developers, being able to see where sites are struggling would allow for the development of strategic methods for improving their support. For researchers, such measures of process are needed to begin testing the efficacy of existing implementation approaches and frameworks.

### Prediction

The expansion of the SIC will allow for the evaluation of site implementation behavior through observation of progress (both time to completion of key tasks and proportion of tasks completed), thereby providing fine-grained data on progress toward the attainment of key implementation objectives. Such data can serve as ‘milestone implementation outcomes’ in and of themselves. In the proposed study, achievement of these outcomes will be observed within and across the practices being studied. At each stage, data on time to completion and proportion of tasks completed during previous stages can be used to predict future milestone achievement at later stages. This data will be considered in relation to qualitative data collected from end-users to increase understanding of the underlying mechanisms assessed by SIC scores. This type of fine-grained data will allow for an examination of which implementation activities are crucial for program success and which might be encouraged but are not essential.

### Cost

When decision or policy makers consider whether or not to implement a new EBP, they must consider not only the cost of the intervention, but also the cost of implementation. They must decide preemptively whether or not to invest in a new practice. This can be a daunting task; implementation costs are likely to differ not only across EBPs, but also between different implementation strategies [19]. Such opportunity costs must be considered against the uncertainty of future benefits. Knowing when different types of costs can be expected during the implementation process could prove critical in helping decision makers map out a clear fiscal plan to ensure proper and timely resource allocation. Data on cost per stage could potentially be reassuring for sites and could clarify what resources are needed, decreasing the potential for both under- and overestimation of resource needs. For example, if a site has struggled to complete certain implementation activities and is unclear if they should proceed, knowing the resource allocation necessary for the next set of implementation activities within the current fiscal year might be beneficial in making this decision. In addition, having a standardized method of assessing implementation costs will allow for future economic evaluations of implementation strategies.

### Impact

Outcomes ideally will help pave the way for future studies on the development of interventions to improve implementation approaches, ultimately leading to more successful EBP implementations in child public service systems and increased availability of EBPs in usual care settings. Study findings will be used to help inform policy makers and other decision makers on what steps and resources are necessary for successful implementation efforts. Moreover, identification of universal implementation activities across EBPs will inform understanding of key variables necessary across implementations to more adequately prepare for implementation success. This knowledge will help increase understanding of ‘what it takes’ to install EBPs in real-world settings and, consequently, increase the availability of the most beneficial services to clients and decrease wasted efforts and resources on failed implementation attempts.

## Abbreviations

EBP: Evidence-based practice; SIC: Stages of implementation completion; MTFC: Multidimensional treatment foster care; MST: Multisystemic therapy; CC: Coping CAT; MDFT: Multidimensional family therapy.

## Competing interests

The author declares that she has no competing interests.

## Supplementary Material

Additional file 1 **Appendix A: Table 1.** The MTFC-SIC from the MTFC Implementation Trial: Activity within Pre-Implementation (Pre-Imp), Implementation (Imp), and Sustainability (Sus) Phases. Click here for file

Additional file 2 Appendix B: MTFC-SIC Measurement Properties, Challenges, and Solutions. Click here for file
