# Supplementary material for: The stages of implementation completion for evidence-based practice: protocol for a mixed methods study
Source: Implement Sci. 2014 Apr 5;9:43. doi: 10.1186/1748-5908-9-43 (PMC4234147; doi:10.1186/1748-5908-9-43)
Supplement: Additional file 1 — Appendix A: Table 1. The MTFC-SIC from the MTFC Implementation Trial: Activity within Pre-Implementation (Pre-Imp), Implementation (Imp), and Sustainability (Sus) Phases. [file 1748-5908-9-43-S1.pdf]

APPENDIX A

Table 1. *The MTFC-SIC from the MTFC Implementation Trial: Activity within Pre-Implementation (Pre-Imp), Implementation (Imp), and Sustainability (Sus) Phases.*

Phase	Stage	Activity	Agent Involved
Pre-Imp	Stage 1: Engagement	Date site informed services/program available (not scored)<br>Date of interest indicated<br>Date agreed to consider implementation	System Leader
Pre-Imp	Stage 2: Consideration of Feasibility	Date of response to first planning contact<br>Date of first meeting/ Feasibility Assessment<br>Date feasibility questionnaire completed	System Leader, Agency
Pre-Imp	Stage 3: Readiness Planning	Date of cost/funding plan review<br>Date of staff sequence, timeline, hire plan review<br>Date of Foster Parent recruitment review<br>Date of referral criteria review<br>Date of communication plan review<br>Date of Meeting #2/ Stakeholder meeting<br>Date written implementation plan completed<br>Date MTFC Service Provider Selected	System Leader, Agency
Imp	Stage 4: Staff Hired & Trained	Date agency checklist completed<br>Date 1st staff hired<br>Date Program Supervisor trained<br>Date clinical training held<br>Date Foster Parent training held<br>Date Expert Consultant assigned to site	Agency, Practitioner
Imp	Stage 5: Adherence Monitoring processes in place	Date Parent Daily Report training held (fidelity measure)<br>Date of 1st program administrator call	Agency, Practitioner
Imp	Stage 6: Services and Consultation Begin	Date of first placement<br>Date of first consult call<br>Date of first clinical meeting video received<br>Date of first foster parent meeting video received	Practitioner, Child/Family
Imp	Stage 7: Ongoing services, consultation, fidelity monitoring and feedback	Dates of site visits (3)<br>Date of implementation review (3)<br>Date of final program assessment	Practitioner, Child/Family
Sus	Stage 8: Competency	Date of certification application<br>Date certified	System Leader, Agency, Practitioner
