# Supplementary material for: The stages of implementation completion for evidence-based practice: protocol for a mixed methods study
Source: Implement Sci. 2014 Apr 5;9:43. doi: 10.1186/1748-5908-9-43 (PMC4234147; doi:10.1186/1748-5908-9-43)
Supplement: Additional file 2 — Appendix B: MTFC-SIC Measurement Properties, Challenges, and Solutions. [file 1748-5908-9-43-S2.pdf]

APPENDIX B

MTFC-SIC MEASUREMENT PROPERTIES, CHALLENGES, AND SOLUTIONS

Measurement properties of the MTFC-SIC were evaluated in preparation for this protocol. Several challenges for evaluating its psychometric properties were considered. The activity completion dates create two types of items: Proportion items are standard dichotomous items (was the activity completed or not?), and Duration items follow a time-distribution (how long did it take to complete the activity?). The Duration item format presents an obstacle for estimating basic properties like internal consistency. There also are challenges around missing data. With multiple stages, activities in uninitiated stages are considered missing (i.e., ineligible). However, such missing data is not readily accommodated by traditional psychometric models. Likewise, the sample size is modest, and most models require large samples. Finally, the data are nested with activities within stages that are nested within sites. Nesting is important to model but not feasible for many traditional psychometric models.

Given these challenges, the reliability and validity of the MTFC-SIC items [24, 25] were evaluated using IRT-based Rasch models [26]. This highly flexible family of models addresses each of the challenges noted above: the “time” distribution, missing data for some activities, modest sample size, and nested data. According to the model, the probability of a site completing an activity is a function of the difficulty of the activity and the implementation level of the site. For example, a site with high adherence would have a high probability of completing a basic activity. Proportion items and Duration items were evaluated using dichotomous and Poisson [27] models [25, 28] and their multilevel extensions (HLM) [29].

Using Rasch modeling, we found that the SIC measurement approach can be validly used for MTFC, increasing the odds of successfully extending the approach to other EBPs. Scores for both activities and sites estimated by the models were found to “match” what experts thought (e.g., completing clinical training is an “easy” activity as everyone had to complete training in

order to implement). There also was sufficient heterogeneity across sites and activities, and there was no evidence of floor or ceiling effects.

Similarly, Rasch reliability statistics of separation for both Duration and Proportion. Activity items demonstrated higher reliability than sites; however, both were in the acceptable range. This indicates adequate reliability of the MTFC-SIC, and that the measurement approach can make the distinctions necessary for evaluating implementation. Finally, an analysis of proportion items suggested no risk of item misfit, whereas several Duration items suggested misfit. The misfitting activities were mandatory in nature, producing inconsistent “difficulty” estimates. This provides evidence that the SIC approach can be defined and implemented such that it performs in the manner expected.

NESTING EFFECTS. Nesting effects were evaluated for activities nested within stages that were nested within sites using Bernoulli and Poisson distributions. Outcomes indicated that the SIC measures features of both stages and sites, the nesting effects are strong enough that they need to be addressed in the models, and the effects can be efficiently modeled using a multilevel Rasch model.
